# Cysteinyl Leukotriene Receptor Antagonists Decrease Cancer Risk in Asthma Patients

**DOI:** 10.1038/srep23979

**Published:** 2016-04-07

**Authors:** Ming-Ju Tsai, Ping-Hsun Wu, Chau-Chyun Sheu, Ya-Ling Hsu, Wei-An Chang, Jen-Yu Hung, Chih-Jen Yang, Yi-Hsin Yang, Po-Lin Kuo, Ming-Shyan Huang

**Affiliations:** 1 Division of Pulmonary and Critical Care Medicine, Department of Internal Medicine, Kaohsiung Medical University Hospital, Kaohsiung Medical University, Kaohsiung, Taiwan; 2 Graduate Institute of Medicine, College of Medicine, Kaohsiung Medical University, Kaohsiung, Taiwan; 3 Division of Nephrology, Department of Internal Medicine, Kaohsiung Medical University Hospital, Kaohsiung Medical University, Kaohsiung, Taiwan; 4 Institute of Clinical Medicine, College of Medicine, Kaohsiung Medical University, Kaohsiung, Taiwan; 5 Department of Internal Medicine, School of Medicine, College of Medicine, Kaohsiung Medical University, Kaohsiung, Taiwan; 6 Department of Internal Medicine, Kaohsiung Municipal Ta-Tung Hospital, Kaohsiung Medical University, Kaohsiung, Taiwan; 7 School of Pharmacy, College of Pharmacy, Kaohsiung Medical University, Kaohsiung, Taiwan; 8 Division of Geriatrics and Gerontology, Department of Internal Medicine, Kaohsiung Medical University Hospital, Kaohsiung Medical University, Kaohsiung, Taiwan

## Abstract

Previous *in vitro* and *in vivo* studies have demonstrated the potential of using cysteinyl leukotriene receptor antagonists (LTRAs) for chemoprevention, but this has not been investigated in any clinical setting. We therefore investigated the chemopreventive effect of LTRAs in a nationwide population-based study. From the Taiwan National Health Insurance Research Database, we enrolled adults with newly-diagnosed asthma between 2001 and 2011. Among these patients, each LTRA user was matched with five randomly-selected LTRA non-users by sex, age, asthma diagnostic year and modified Charlson Comorbidity Index score. We considered the development of cancer as the outcome. Totally, 4185 LTRA users and 20925 LTRA non-users were identified. LTRA users had a significantly lower cancer incidence rate than LTRA non-users did. Multivariable Cox regression analyses adjusting for baseline characteristics and comorbidities showed LTRA use was an independent protecting factor (hazard ratio = 0.31 [95% CI: 0.24–0.39]), and cancer risk decreased progressively with higher cumulative dose of LTRAs. In conclusion, this study revealed that the LTRA use decreased cancer risk in a dose-dependent manner in asthma patients. The chemopreventive effect of LTRAs deserves further study.

Cancer is a leading cause of death worldwide and has become the most common cause of death in Taiwan for more than 25 years^1^. Although much improvement has been made in anti-cancer treatment, the therapeutic outcome remained unsatisfying. Developing preventive strategies to reduce cancer incidence is therefore as important as improving anti-cancer strategies^2^,^3^. Chemoprevention is the use of a specific agent to reverse, suppress, or prevent the process of carcinogenesis^2^,^3^,^4^. Because limited effective and potent chemopreventive strategies are available to date, the cancer incidence remained high. Taking lung cancer, the most common cause of cancer death, for example, no specific agents have been recommended for primary, secondary, or tertiary chemoprevention although much effort has been made in the field of chemoprevention research^4^.

Cysteinyl leukotriene receptor antagonists (LTRAs), such as montelukast and zafirlukast, are widely used drugs for treating allergic asthma^5^,^6^. In addition to its well-known role in asthma, the leukotriene pathway is also responsible for carcinogenesis and tumour-mediated immunosuppression^7^. Overexpression of a cysteinyl leukotriene receptor, CysLT_1_R, has been shown in colorectal cancer, prostate cancer, renal cell carcinoma, transitional cell carcinoma and testicular cancer, and montelukast induces apoptosis of these cancer cells^8^,^9^,^10^,^11^,^12^,^13^,^14^. Only few *in vivo* studies to date have reported the chemopreventive effect of leukotriene pathway inhibitors^14^,^15^,^16^, while the chemopreventive effect of LTRAs has not been investigated in clinical setting.

Because some *in vitro* and *in vivo* studies had demonstrated the potential of using LTRAs for chemoprevention, we therefore conducted a nationwide population-based study to investigate the chemopreventive effect of LTRAs. Using a retrospective cohort study design, we found that LTRA use was associated with a decreased cancer risk in a dose-dependent manner.

## Methods

### Data Source

The Taiwan National Health Insurance (NHI) has covered ambulatory care, inpatient care and prescription drugs in Taiwan since 1996. The NHI coverage rate was 96.2% of whole population in 2000 and increased to >99% by 2005^2^,^17^,^18^,^19^,^20^,^21^. The NHI Research Database therefore comprises comprehensive health care information from nearly the entire population of 23.72 million in Taiwan, becoming one of the largest insurance databases in the world^17^,^19^,^20^,^21^,^22^,^23^. The database used for this study is a cohort of two million subjects randomly sampled from NHI beneficiaries in 2000, and has been verified to be representative of the overall population of beneficiaries in terms of age, sex, geographic distribution and healthcare costs. The database includes information on medical reimbursement claims (such as ambulatory care claims, inpatient care claims, prescriptions, and registration entries) as well as information from Catastrophic Illness Registry, National Cancer Registry and National Register of Deaths. The database is managed by the Collaboration Center of Health Information Application (CCHIA), Ministry of Health and Welfare. For protection of confidentiality, patient identification has been already encrypted, and the authorized researchers are only permitted to perform data linkage, processing and statistical analyses with a specified computer in a closely monitored room. Using the scrambled personal identifier for each subject, the researchers are able to link the files to obtain socio-demographic information, longitudinal medical history and other information. Only statistical results were allowed to be brought out.

### Study population

**Figure 1:**
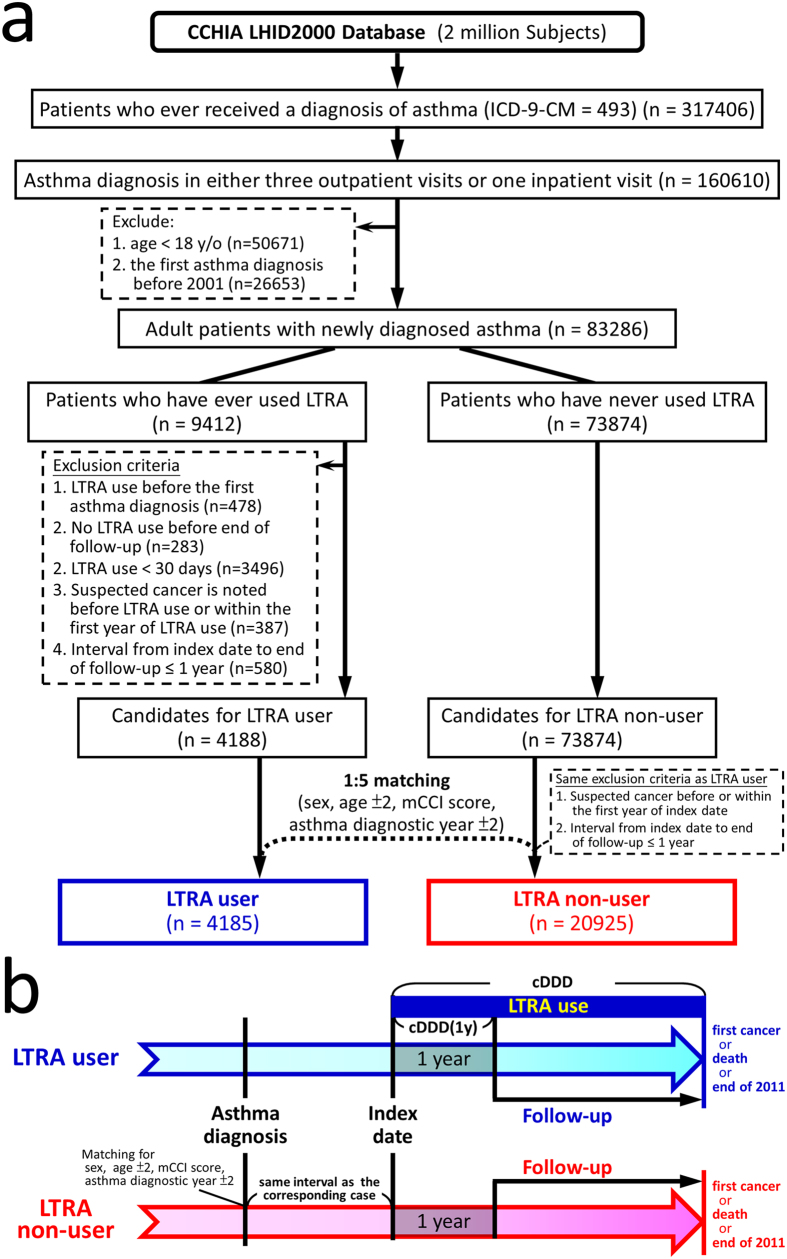
(**a**) Algorism for identifying the study cohorts. (**b**) Study design. From the dataset, adult patients with newly diagnosed asthma were identified. Through the algorism, subjects using LTRA for more than a month (30 days) before the end of follow-up were identified as candidates for LTRA user cohort. The subjects who had never used LTRA were identified as candidates for LTRA non-user cohort. Each LTRA user was matched with five randomly-selected LTRA non-users by sex, age (±2), asthma diagnostic year (±2) and mCCI score. The index date was defined as the date of first LTRA prescription for LTRA users; the LTRA non-users were given the index date with the same interval from their first asthma diagnosis as their corresponding LTRA users. During the matching process, the same exclusion criteria for the LTRA users were also applied while selecting LTRA non-users to ensure enough follow-up time and absence of any cancer diagnosis before the end of the first year after index date. The subjects were followed from a year after the index date to either development of cancer, death or the end of 2011, whichever came first. The cumulative defined daily doses of LTRA were calculated from the index date to the end of follow-up (cDDD) and to a year after the index date [cDDD(1y)]. Abbreviations: CCHIA = Collaboration Center of Health Information Application; LHID = Longitudinal Health Insurance Database; ICD-9-CM = International Classification of Diseases, Ninth Revision, Clinical Modification code; mCCI = modified Charlson Comorbidity Index.

From the dataset, patients with newly diagnosed asthma were identified by the algorithm showed in Fig. 1. Patients with asthma diagnosis (International Classification of Diseases, Ninth Revision, Clinical Modification code [ICD-9-CM] of 493) in the ambulatory or inpatient claim database were identified, and only those with asthma diagnosis in at least three ambulatory claims or one inpatient claim were enrolled^18^. To ensure newly diagnosed adult asthma, those having asthma diagnosis before 2001 or those younger than 18 years old on their first asthma diagnosis were excluded.

The subjects who had ever used either montelukast or zafirlukast, the LTRAs available in Taiwan, after their asthma diagnoses were identified. After excluding those with neoplasm diagnosis (ICD-9-CM of 140-239 in any claims) before the end of the first year of LTRA use and those with the interval between first LTRA prescription and end of follow-up ≤1 year, subjects using LTRA for ≥30 days before the end of follow-up were identified as candidates for LTRA user cohort. The subjects who had never used LTRA were identified as candidates for LTRA non-user cohort.

### Definitions of variables

The endpoint of this study was the development of cancer, defined by the appearance of cancer diagnosis in Catastrophic Illness Registry or National Cancer Registry. Pathological confirmation is generally required for reporting a cancer diagnosis to these registries. The date of death was obtained from the National Register of Deaths.

The presence of comorbidity was identified by the presence of any corresponding diagnostic codes before the index date in the claim databases and confirmed by the presence of the codes for at least three times in the ambulatory claim database or at least once in the inpatient claim database. Based on the comorbidities, modified Charlson Comorbidity Index (mCCI) score was calculated by subtracting chronic pulmonary disease from the original Charlson Comorbidity Index score^24^.

### Study cohorts

Each LTRA user was matched with five randomly-selected LTRA non-users by sex, age (±2), asthma diagnostic year (±2) and mCCI score. The index date was defined as the date of first LTRA prescription for LTRA users; the LTRA non-users were given the index date with the same interval from their first asthma diagnosis as their corresponding LTRA users. During the matching process, the same exclusion criteria for the LTRA users were also applied while selecting LTRA non-users to ensure enough follow-up time and absence of any cancer diagnosis before the end of the first year after index date.

To minimize immortal time bias, the follow-up period was calculated from a year after the index date. The subjects were followed from a year after the index date to either development of cancer, death or the end of 2011, whichever came first. The defined daily doses (DDD) were 10 mg and 40 mg for montelukast and zafirlukast, respectively. To quantify individual’s exposure to LTRA, the cumulative defined daily doses of LTRA from the index date to the end of follow-up (cDDD) and to a year after the index date [cDDD(1y)] were calculated.

### Statistical analysis

The demographic data and comorbidities were compared between LTRA users and non-users using Pearson’s χ^2^ test for categorical variables or Student’s t test for continuous variables, as appropriate. The cancer incidence rate (IR) was calculated as the number of cancer developed during the follow-up period divided by the total person-year. The cancer IRs in LTRA users and non-users were compared by estimating the incidence rate ratio (IRR) using Poisson regression and adjusted IRR (aIRR) using multivariable Poisson regression after adjusting for age, residency, income level, marriage status, education level and the presence of various comorbidities. Cumulative incidence of cancer was calculated and compared with Kaplan-Meier method and log-rank test. To further assess the effect of LTRA, multivariable Cox proportional hazards regression analyses were performed with adjustment of the same covariates as in Poisson regression. In addition, stratified analyses were also performed for Poisson and Cox regression in subgroups of covariates. To determine the effect of LTRA on the risk of different cancers, we also calculated the hazard ratios of LTRA use for several major cancers in Taiwan.

Extraction and computation of data, data linkage, processing and sampling and statistical analyses were performed using SAS system (version 9.3 for Windows, SAS Institute Inc., Cary, NC). The statistical significance level was set at a two-sided *p* value of <0.05.

## Results

From the database, 317406 asthma patients were identified. Through the algorithm (Fig. 1), 4185 LTRA users and 20925 matched LTRA non-users were identified. The mean (±SD) age was 47.3 (±16.5) years, and 59% of the subjects were female (Table 1). LTRA users had significantly higher income and higher education level as compared with LTRA non-users, and more LTRA users lived in northern Taiwan. In the LTRA users, 3975 (95%) and 366 (9%) subjects had ever used montelukast and zafirlukast, respectively; the median (IQR) of cDDD and cDDD(1y) were 101 (56–235) and 77 (42–145), respectively.

**Figure 2:**
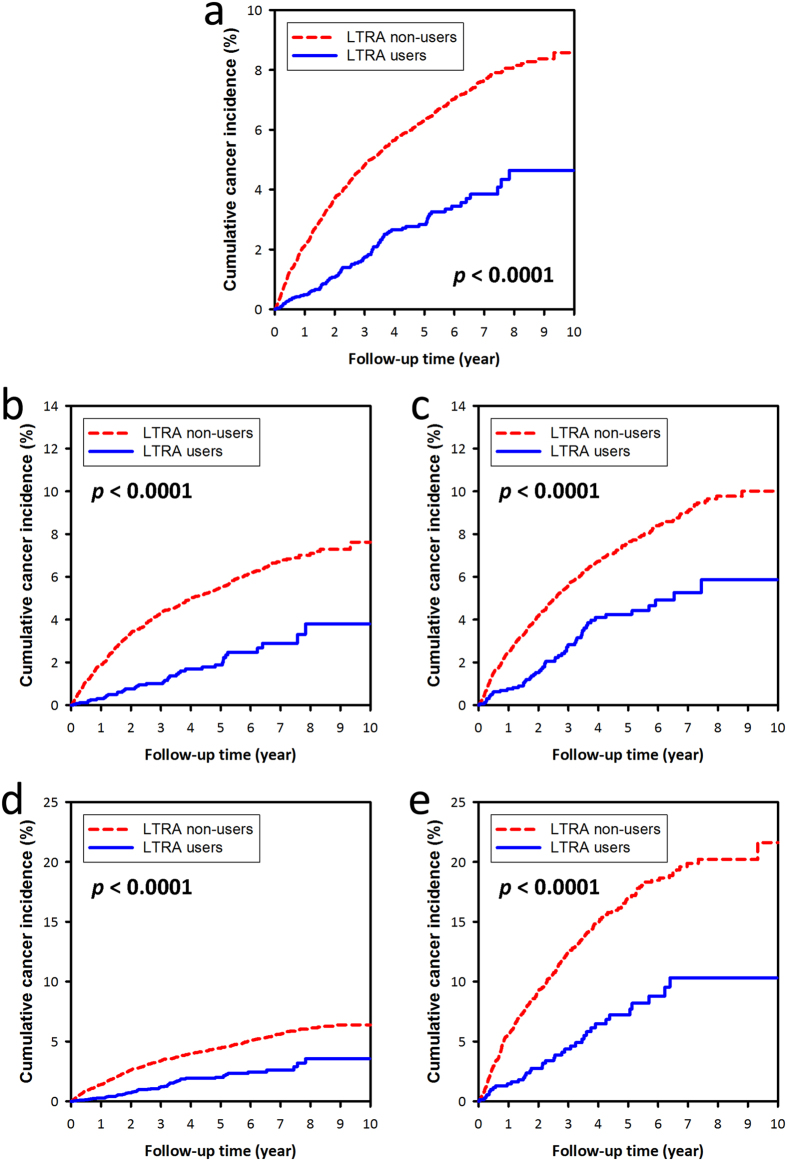
The cumulative cancer incidence of (**a**) the whole study population, (**b**) female patients, (**c**) male patients, (**d**) subjects ≤65 years old, and (**e**) subjects >65 years old. The red dashed lines and blue continuous lines show the cumulative cancer incidence of LTRA non-users and LTRA users, respectively. LTRA users had a significantly lower cumulative cancer incidence than LTRA non-users did (*p* < 0.0001).

LTRA users had a significantly lower cancer IR than LTRA non-users did (5.8 vs. 13.1 per 1000 patient-years; aIRR = 0.41 [95% CI: 0.36–0.47], *p* < 0.0001) (Table 2), and all stratified analyses showed consistent findings. The cumulative cancer incidence was significantly lower in LTRA users than in LTRA non-users (*p* < 0.0001) (Fig. 2a). On stratified analyses, the LTRA users had a significantly lower cumulative cancer incidence as compared with LTRA non-users in strata of female, male, younger and elder subjects (all *p* < 0.0001) (Fig. 2b–e).

**Figure 3:**
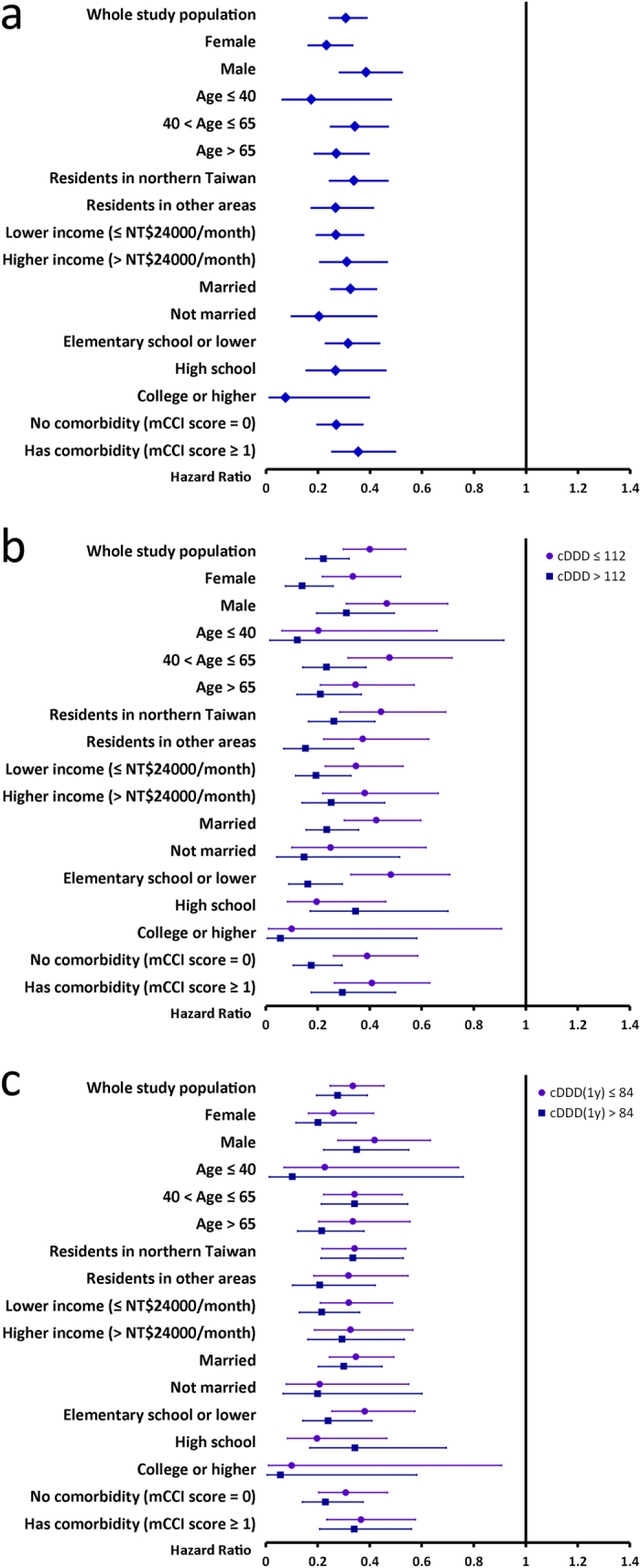
Stratified analyses of the multivariable Cox proportional hazards regression analyses showing adjusted hazard ratios (HRs) of (**a**) LTRA use and (**b,c**) lower and higher doses of LTRA use. The results are presented with adjusted HRs (95% confidence interval) of either (**a**) LTRA use or (**b,c**) lower and higher doses of LTRA use, which are adjusted for age, residency, income level, marriage status, education level and the presence of various comorbidities (except for the variable used for stratification). The follow-up time was calculated from a year after the index date to either development of cancer, death or the end of 2011, whichever came first. The cumulative defined daily doses of LTRA were calculated from the index date to the end of follow-up (cDDD) and to a year after the index date [cDDD(1y)].

On multivariable Cox proportional hazards regression analyses adjusting for age, residency, income level, marriage status, education level and comorbidities, LTRA use was associated with a decreased cancer risk (hazard ratio = 0.31 [95% CI: 0.24–0.39], *p* < 0.0001) (Table 3, model 1). The cancer risk decreased progressively with higher cumulative dose of LTRA use as compared with LTRA non-users. LTRA users with lower and higher cDDD of LTRA had 60% and 78% cancer risk reduction, respectively (Table 3, model 2). Similarly, LTRA users with lower and higher cDDD(1y) of LTRA had a 66% and 72% cancer risk reduction, respectively (Table 3, model 3). On stratified analyses, LTRA use was associated with a significantly lower cancer risk in all strata (Fig. 3a). LTRA users with higher cDDD or cDDD(1y) use had lower cancer risk than those with lower cDDD or cDDD(1y) did in nearly all strata (Fig. 3b,c). The significant effect of LTRA on cancer risk reduction was observed mainly in lung, colorectal, liver and breast cancer (Table 4).

## Discussion

This large population-based study revealed that LTRA use was associated with a decreased cancer risk in asthma patients. Particularly, the chemopreventive effect appeared larger with a higher cumulative dose, indicating a dose-dependent manner of LTRA in this issue. The strengths of this study are its population-based sampling, avoidance of selection bias, adjustment for confounders, and, most importantly, the demonstration of dose-dependent protection effect. To the best of our knowledge, we are not only the first to report the chemopreventive effect of LTRAs in the clinical setting but also the first to demonstrate a dose-response relationship between the use of LTRAs and reduced risk of cancer. Further clinical studies are required to confirm our findings, and further *in vivo* and *in vitro* studies should be taken to investigate the chemopreventive mechanisms of LTRAs.

As inflammation is a major contributor for carcinogenesis and cancer progression, immune responses are the most important mechanisms running in tumour microenvironment. Indeed, the interaction between cancer cells and the surrounding immune cells have been noted to form a milieu which is suitable for carcinogenesis, as well as proliferation and migration of cancer cells^25^. Eicosanoids involve in a variety of inflammatory and immune responses throughout the body, and are also important regulators in the immune responses in tumour microenvironment^7^. Using selective cyclooxygenase-2 (COX-2) inhibitors for chemoprevention is therefore widely discussed. Our previous population-based study indicated that selective COX-2 inhibitors reduced development of colorectal cancer by at least 10%^3^.

In recent years, the role of leukotriene pathway in carcinogenesis and tumour-mediated immunosuppression has been increasingly recognized^7^,^26^. While much effort has been made in identifying the role of LTB_4_ pathway in cancer, the tumour-promoting role of cysteinyl leukotrienes, including LTC_4_, LTD_4_ and LTE_4_, is less studied. Cysteinyl leukotrienes are originally recognized for their effect to promote bronchoconstriction, inflammation, microvascular permeability and mucus secretion^5^. Since more than a decade ago, LTD_4_ has been shown to reduce apoptosis, enhance proliferation, induce transcriptional activity of potentially oncogenic genes and induce migration of intestinal epithelial cells^27^. Clinically, increased expression of CysLT_1_R was noted in specimens from colorectal, gastric and breast cancers, and the elevated CysLT_1_R expression correlated to poorer survival^13^,^28^,^29^,^30^. The circulating LTD_4_ level was significantly higher in patients with hepatocellular carcinoma than in healthy subjects^31^. Over-expression of CysLT_1_R has also been shown in prostate cancer, renal cell carcinoma, transitional cell carcinoma and testicular cancer, and montelukast induces early apoptosis of these cancer cells^8^,^9^,^10^,^11^,^12^,^14^.

In addition to the pro-apoptotic effect of montelukast on few cancer cell lines, however, only few *in vivo* studies have reported chemoprevention effect of leukotriene pathway inhibitors in the literature while no clinical study is available currently. An early study demonstrated chemopreventive effect of leukotriene pathway inhibitors, accolate, zileuton and MK-866, in vinyl carbamate-induced lung tumours in mice^15^. In an *in vivo* LLC cells metastasis model, pranlukast and montelukast prevented tumour metastasis through peripheral capillaries^16^. A recent study using nude mice demonstrated that an LTRA, ZM198,615 or montelukast, inhibited the growth of colon cancer xenografts^14^.

In contrast to our previous study showing about 50% cancer risk reduction in users of selective COX-2 inhibitor, the present study showed an impressive 60–78% cancer risk reduction with using LTRAs^3^. Many adverse effects of selective COX-2 inhibitors, especially renal failure and cardiovascular complications, prevent their wide application. LTRAs used in current clinical practice are generally so safe that can be used in paediatric asthma patients^6^. After our findings are further validated in other clinical studies, using LTRAs for chemoprevention might be much easier due to their satisfying safety profiles.

There are several limitations in our studies. First, some well-known potentially important clinical covariates, such as smoking history and environmental exposure, are not available in the database. Interpreting of our results must be careful to account for possible impacts of these risk factors. Nevertheless, LTRA users and LTRA non-users are matched by sex, age, asthma diagnostic year and mCCI score, and Cox regression analyses were adjusted for age, residency, income level, marriage status, education level and comorbidities. To address the potential issues caused by the administrative database, we also conducted various stratified analyses and found consistent results. Because smoking rate was much lower in female (4.2%) than in male (46.8%) in Taiwan^2^,^32^, the results of stratified analyses in female subjects might be taken as a proxy for the effect of LTRAs in non-smokers. In this study, the chemopreventive effect of LTRAs seemed more pronounced in the female and younger subjects, as compared to male subjects and elder subjects, respectively. These findings suggested that the chemopreventive effect of LTRAs might be more pronounced in non-smokers than in smokers, and the detailed mechanisms deserves further study. Second, because our studies enrolled only asthma patients, whether the results can be applied to patients without asthma needs further study. However, because LTRAs are mainly used for allergic asthma, choosing subjects from asthma patients are therefore required to homogenize the case and control cohorts. Third, time-related biases were always a concern as in many observational studies^33^. Our study design inherently avoided time-lagging bias by unifying the interval between the initial asthma diagnosis and the index date of an LTRA non-user with that of the corresponding LTRA user. Although immortal time bias and time-window bias were not totally avoided in this study, our study design substantially minimized the impact of these biases. Besides, asthma was not associated with significantly increased cancer incidence^17^. Furthermore, the dose-dependent effect shown in multivariable Cox regression analyses increased the reliability of our results. Finally, this study was conducted in patients of Han Chinese ethnicity. Whether the findings are also applicable to other ethnic population require further evaluation.

In summary, our study reveals that the use of LTRA in asthma patients is associated with a decreased risk of cancer in a dose-dependent manner. The utility of LTRAs as chemopreventive agents deserves further study in depth.

## Additional Information

**How to cite this article**: Tsai, M.-J. *et al*. Cysteinyl Leukotriene Receptor Antagonists Decrease Cancer Risk in Asthma Patients. *Sci. Rep.* **6**, 23979; doi: 10.1038/srep23979 (2016).

## Figures and Tables

**Table 1:** Baseline characteristics of the study population.

	All patients (n = 25110)	LTRA non-users (n = 20925)	LTRA users (n = 4185)	*P* value
Sex, n (%)#				
Female	14934 (59%)	12445 (59%)	2489 (59%)	
Male	10176 (41%)	8480 (41%)	1696 (41%)	
Age (year), mean ± SD#	47.3 ± 16.5	47.3 ± 16.5	47.2 ± 16.7	0.6696
Age (year), n (%)#				0.5387
Age ≤40	9559 (38%)	7936 (38%)	1623 (39%)	
40 <Age ≤65	11061 (44%)	9247 (44%)	1814 (43%)	
Age >65	4490 (18%)	3742 (18%)	748 (18%)	
Interval between asthma diagnosis to index date (year), median (IQR)#	0.8 (0–3.4)	0.8 (0–3.4)	0.8 (0–3.4)	
Residency, n (%)				<0.0001
Northern Taiwan	13836 (55%)	11191 (53%)	2645 (63%)	
Other areas	11274 (45%)	9734 (47%)	1540 (37%)	
Monthly income (NT$), median (IQR)	25200 (21900–42000)	25200 (21900–42000)	27600 (21900–43900)	<0.0001
Monthly income (NT$), n (%)				<0.0001
≤24000	12290 (49%)	10412 (50%)	1878 (45%)	
>24000	12820 (51%)	10513 (50%)	2307 (55%)	
Marriage status, n (%)				0.2194
Married	16049 (64%)	13409 (64%)	2640 (63%)	
Not married	9061 (36%)	7516 (36%)	1545 (37%)	
Education level, n (%)				<0.0001
Elementary school or lower	9616 (38%)	8131 (39%)	1485 (35%)	
High school	11136 (44%)	9310 (44%)	1826 (44%)	
College or higher	4358 (17%)	3484 (17%)	874 (21%)	
With comorbidity, n (%)#				
No (mCCI score = 0)	21630 (86%)	18025 (86%)	3605 (86%)	
Yes (mCCI score ≥1)	3480 (14%)	2900 (14%)	580 (14%)	
Comorbidity, n (%)				
Heart disease	979 (4%)	814 (4%)	165 (4%)	0.8726
Myocardial infarction	145 (1%)	123 (1%)	22 (1%)	0.6282
Congestive heart failure	878 (3%)	726 (3%)	152 (4%)	0.6014
Peripheral vascular disease	185 (1%)	144 (1%)	41 (1%)	0.0441
Major neurological disorder	1582 (6%)	1342 (6%)	240 (6%)	0.0991
Cerebral vascular disease	1520 (6%)	1295 (6%)	225 (5%)	0.0442
Dementia	160 (1%)	130 (1%)	30 (1%)	0.4781
Hemiplegia	118 (0%)	100 (0%)	18 (0%)	0.6799
Connective tissue disease	393 (2%)	319 (2%)	74 (2%)	0.2462
Peptic ulcer disease	4845 (19%)	4015 (19%)	830 (20%)	0.3343
Liver disease	2449 (10%)	2006 (10%)	443 (11%)	0.0468
Diabetes mellitus	2018 (8%)	1698 (8%)	320 (8%)	0.3090
Renal disease	479 (2%)	401 (2%)	78 (2%)	0.8205

Categorical variables and continuous variables were compared using χ^2^ test and Student’s t-test, respectively.

Abbreviation: LTRA = cysteinyl leukotriene receptor antagonist; SD = standard deviation; IQR = interquartile range; NT = New Taiwan Dollar; mCCI = modified Charlson Comorbidity Index.

^#^matched factors.

**Table 2:** Incidence rates of cancer in LTRA users and non-users.

	All patients				LTRA non-users			IR	LTRA users				IRR [95% CI]	aIRR [95% CI]
	N	Cancer	PY	IR	N	Cancer	PY		N	Cancer	PY	IR		
**Whole study population Stratified analyses**	25110	1197	100593.2	11.9	20925	1104	84593.3	13.1	4185	93	15999.9	5.8	0.45 [0.39–0.51]^***^	0.41 [0.36–0.47]^***^
Sex														
Female	14934	625	61015.7	10.2	12445	585	51404.0	11.4	2489	40	9611.7	4.2	0.37 [0.30–0.44]^***^	0.34 [0.28–0.41]^***^
Male	10176	572	39577.5	14.5	8480	519	33189.3	15.6	1696	53	6388.2	8.3	0.53 [0.44–0.64]^***^	0.49 [0.41–0.58]^***^
Age														
Age ≤40	9559	109	43348.8	2.5	7936	104	36702.5	2.8	1623	5	6646.3	0.8	0.27 [0.19–0.36]^***^	0.27 [0.20–0.37]^***^
40< Age ≤65	11061	620	43523.3	14.2	9247	570	36702.9	15.5	1814	50	6820.4	7.3	0.47 [0.39–0.57]^***^	0.45 [0.37–0.55]^***^
Age >65	4490	468	13721.1	34.1	3742	430	11187.9	38.4	748	38	2533.2	15.0	0.39 [0.30–0.51]^***^	0.38 [0.29–0.50]^***^
Residency														
Northern Taiwan	13836	589	56174.4	10.5	11191	530	46127.2	11.5	2645	59	10047.2	5.9	0.51 [0.43–0.60]^***^	0.43 [0.37–0.50]^***^
Other areas	11274	608	44418.8	13.7	9734	574	38466.1	14.9	1540	34	5952.7	5.7	0.38 [0.30–0.48]^***^	0.38 [0.30–0.47]^***^
Monthly income														
≤NT$24000	12290	759	47025.8	16.1	10412	708	39915.9	17.7	1878	51	7109.9	7.2	0.40 [0.33–0.49]^***^	0.36 [0.30–0.44]^***^
>NT$24000	12820	438	53567.4	8.2	10513	396	44677.4	8.9	2307	42	8890.0	4.7	0.53 [0.45–0.64]^***^	0.48 [0.41–0.57]^***^
Marriage status														
Married	16049	921	64873.1	14.2	13409	845	54554.7	15.5	2640	76	10318.4	7.4	0.48 [0.41–0.56]^***^	0.44 [0.38–0.51]^***^
Not married	9061	276	35720.1	7.7	7516	259	30038.6	8.6	1545	17	5681.5	3.0	0.35 [0.27–0.45]^***^	0.31 [0.24–0.39]^***^
Education level														
Elementary school or lower	9616	731	34513.9	21.2	8131	680	29182.4	23.3	1485	51	5331.6	9.6	0.41 [0.33–0.51]^***^	0.39 [0.32–0.48]^***^
High school	11136	348	47369.8	7.3	9310	319	40247.0	7.9	1826	29	7122.8	4.1	0.51 [0.42–0.63]^***^	0.43 [0.35–0.52]^***^
College or higher	4358	118	18709.5	6.3	3484	105	15163.9	6.9	874	13	3545.6	3.7	0.53 [0.39–0.71]^***^	0.42 [0.32–0.55]^***^
With comorbidity														
No (mCCI score = 0)	16500	685	71023.6	9.6	13750	636	59945.8	10.6	2750	49	11077.8	4.4	0.42 [0.35–0.50]^***^	0.38 [0.32–0.44]^***^
Yes (mCCI score ≥ 1)	8610	512	29569.6	17.3	7175	468	24647.5	19.0	1435	44	4922.1	8.9	0.47 [0.38–0.58]^***^	0.45 [0.37–0.55]^***^

The subjects were followed from a year after the index date to either development of cancer, death or the end of 2011, whichever came first.

The incidence rate (IR) is expressed as incident cancer per 1000 patient-years. The IRs of cancer in LTRA users and non-users were compared by estimating the incidence rate ratio (IRR) using Poisson regression and adjusted IRR (aIRR) using multivariable Poisson regression after adjusting for age, residency, income level, marriage status, education level and the presence of various comorbidities (except for the variable used for stratification).

^*^P < 0.05; ^**^P < 0.01; ^***^P < 0.0001.

Abbreviation: N = number of patients; Cancer = number of patients with incident cancer; PY = total patient-years of follow-up; CI = confidence interval.

**Table 3:** Multivariable Cox regression analyses of the related factors for developing cancer in asthma patients.

	Model 1				Model 2				Model 3			
	HR	95% CI		*P* value	HR	95% CI		*P* value	HR	95% CI		*P* value
		lower	upper			lower	upper			lower	upper	
Age: (vs. age ≤40)												
40< Age ≤65	0.74	0.30	1.80	0.5049	0.76	0.31	1.85	0.5460	0.74	0.30	1.80	0.5046
Age >65	1.24	0.44	3.50	0.6847	1.29	0.46	3.60	0.6325	1.24	0.44	3.50	0.6840
Residency (northern Taiwan vs. other areas)	1.02	0.88	1.17	0.8385	1.02	0.89	1.17	0.7706	1.02	0.88	1.17	0.8216
Monthly income (>NT$24000 vs. ≤NT$24000)	0.91	0.78	1.07	0.2731	0.92	0.78	1.08	0.2876	0.92	0.78	1.07	0.2780
Marriage status (married vs. not married)	1.06	0.90	1.25	0.4926	1.06	0.90	1.25	0.4873	1.06	0.90	1.25	0.4979
Education level: (vs. elementary school or lower)												
High school	1.00	0.84	1.19	0.9767	1.00	0.84	1.18	0.9561	1.00	0.84	1.19	0.9714
College or higher	1.25	0.96	1.62	0.0934	1.25	0.96	1.62	0.0941	1.25	0.96	1.62	0.0957
Presence of comorbidity:												
Heart disease	1.05	0.68	1.63	0.8126	1.06	0.68	1.64	0.7996	1.06	0.68	1.63	0.8113
Peripheral vascular disease	0.90	0.41	1.99	0.7972	0.89	0.41	1.96	0.7779	0.90	0.41	1.99	0.8027
Major neurological disorder	0.94	0.62	1.43	0.7683	0.94	0.62	1.44	0.7776	0.94	0.62	1.44	0.7778
Connective tissue disease	0.86	0.47	1.57	0.6234	0.85	0.47	1.56	0.6092	0.86	0.47	1.57	0.6225
Peptic ulcer disease	1.12	0.78	1.59	0.5491	1.12	0.78	1.60	0.5354	1.12	0.78	1.60	0.5441
Liver disease	1.56	1.08	2.25	0.0180	1.58	1.09	2.27	0.0153	1.57	1.08	2.26	0.0171
Diabetes mellitus	1.03	0.67	1.57	0.9106	1.02	0.67	1.57	0.9215	1.02	0.67	1.57	0.9162
Renal disease	1.09	0.56	2.13	0.8017	1.09	0.56	2.12	0.8001	1.09	0.56	2.12	0.8013
LTRA users (vs. LTRA non-users)	0.31	0.24	0.39	<0.0001								
cDDD of LTRA (vs. LTRA non-users)												
cDDD ≤112					0.40	0.30	0.54	<0.0001				
cDDD >112					0.22	0.16	0.32	<0.0001				
cDDD(1y) of LTRA (vs. LTRA non-users)												
cDDD(1y) ≤84									0.34	0.25	0.45	<0.0001
cDDD(1y) >84									0.28	0.20	0.39	<0.0001

The follow-up time was calculated from a year after the index date to either development of cancer, death or the end of 2011, whichever came first. The cumulative defined daily doses of LTRA were calculated from the index date to the end of follow-up (cDDD) and to a year after the index date [cDDD(1y)].

Using LTRA non-users as reference, the adjusted HRs of LTRA use (model 1), lower and higher cDDD (model 2) and lower and higher cDDD(1y) were calculated by the multivariable Cox proportional hazards regression analyses adjusted for age, residency, income level, marriage status, education level and the presence of various comorbidities.

Abbreviations: HR = hazard ratio; CI = confidence interval.

**Table 4:** Multivariable Cox regression analyses of the related factors for developing various cancers in asthma patients.

	Model 1		Model 2				Model 3			
	LTRA users		cDDD ≤112		cDDD >112		cDDD(1y) ≤84		cDDD(1y) >84	
	HR [95% CI]	*P* value	HR [95% CI]	*P* value	HR [95% CI]	*P* value	HR [95% CI]	*P* value	HR [95% CI]	*P* value
Lung cancer	0.34 [0.20–0.60]	0.0002	0.43 [0.21–0.90]	0.0256	0.27 [0.12–0.62]	0.0019	0.32 [0.14–0.72]	0.0057	0.37 [0.18–0.78]	0.0094
Colorectal cancer	0.35 [0.20–0.62]	0.0004	0.43 [0.20–0.93]	0.0324	0.28 [0.12–0.66]	0.0037	0.42 [0.19–0.91]	0.0275	0.29 [0.12–0.68]	0.0045
Gastric cancer	0.30 [0.09–0.99]	0.0486	0.37 [0.08–1.71]	0.2040	0.21 [0.03–1.66]	0.1400	0.38 [0.08–1.72]	0.2087	0.21 [0.03–1.62]	0.1328
Liver cancer	0.34 [0.17–0.69]	0.0027	0.44 [0.18–1.08]	0.0738	0.24 [0.08–0.76]	0.0147	0.47 [0.20–1.10]	0.0806	0.19 [0.05–0.70]	0.0129
Pancreatic cancer	0.26 [0.05–1.44]	0.1220	0.24 [0.02–3.13]	0.2742	0.27 [0.03–2.42]	0.2426	0.20 [0.02–2.42]	0.2068	0.33 [0.03–3.50]	0.3553
Oral cancer	0.35 [0.12–1.01]	0.0519	0.32 [0.07–1.43]	0.1343	0.38 [0.08–1.72]	0.2093	0.32 [0.07–1.43]	0.1345	0.38 [0.08–1.72]	0.2100
Nasopharyngeal carcinoma	0.26 [0.03–2.51]	0.2470	^‡^		^‡^		^‡^		^‡^	
Brain cancer	0.26 [0.03–2.51]	0.9974	^‡^		^‡^		^‡^		^‡^	
Thyroid cancer	0.30 [0.06–1.55]	0.1504	^‡^		^‡^		^‡^		^‡^	
Skin cancer	0.61 [0.15–2.53]	0.4964	0.67 [0.10–4.53]	0.6855	0.54 [0.06–4.79]	0.5797	0.71 [0.10–4.82]	0.7259	0.51 [0.06–4.49]	0.5453
Urinary cancer	0.78 [0.33–1.88]	0.5839	0.94 [0.32–2.77]	0.9112	0.55 [0.11–2.82]	0.4752	0.71 [0.22–2.24]	0.5550	0.92 [0.23–3.70]	0.9049
Breast cancer	0.09 [0.03–0.26]	<0.0001	0.15 [0.04–0.49]	0.0019	0.05 [0.01–0.34]	0.0025	0.09 [0.02–0.36]	0.0008	0.10 [0.02–0.44]	0.0022
Cervical cancer	0.48 [0.18–1.26]	0.1341	0.44 [0.12–1.60]	0.2129	0.52 [0.13–2.09]	0.3608	0.53 [0.17–1.64]	0.2718	0.38 [0.07–2.05]	0.2584
Prostate cancer	0.16 [0.03–0.94]	0.0419	0.19 [0.02–1.7]	0.1372	0.14 [0.01–1.74]	0.1265	0.16 [0.02–1.53]	0.1106	0.17 [0.01–2.34]	0.1873

The results are presented with adjusted hazard ratios (HRs) (95% confidence interval) of LTRA users (model 1) or lower (cDDD ≤ 112 in model 2 and cDDD(1y) ≤ 84 in model 3) and higher (cDDD > 112 in model 2 and cDDD(1y) >84 in model 3) doses of LTRA use, using LTRA non-users as reference, which are adjusted for age, residency, income level, marriage status, education level and the presence of various comorbidities.

The follow-up time was calculated from a year after the index date to either development of the specific cancer, death or the end of 2011, whichever came first.

The cumulative defined daily doses of LTRA were calculated from the index date to the end of follow-up (cDDD) and to a year after the index date [cDDD(1y)].

^‡^The HR of some cancer types could not be estimated due to small sample size.
