# Corrigendum: Cysteinyl Leukotriene Receptor Antagonists Decrease Cancer Risk in Asthma Patients

**DOI:** 10.1038/srep25880

**Published:** 2016-05-12

**Authors:** Ming-Ju Tsai, Ping-Hsun Wu, Chau-Chyun Sheu, Ya-Ling Hsu, Wei-An Chang, Jen-Yu Hung, Chih-Jen Yang, Yi-Hsin Yang, Po-Lin Kuo, Ming-Shyan Huang

Scientific Reports 6: Article number: 23979;10.1038/srep23979 Published online: 04072016; Updated: 05122016

The Acknowledgements section in this Article is incomplete:

“The authors thank the help from the Statistical Analysis Laboratory, Department of Internal Medicine and the Statistical Analysis Laboratory, Department of Medical Research, Kaohsiung Medical University Hospital. This study is based in part on data from the National Health Insurance Research Database provided by the National Health Insurance Administration, Ministry of Health and Welfare and managed by the Collaboration Center of Health Information Application (CCHIA), Ministry of Health and Welfare, Executive Yuan, Taiwan. The interpretation and conclusions contained herein do not represent those of Ministry of Health and Welfare. This work was supported by grants from the Ministry of Science and Technology [MOST 104-2314-B-037-005 and MOST 104-2314-B-037-034-MY3]; and the Aim for the Top Journals Grant, Kaohsiung Medical University Research Foundation [KMU-DT103008].”

should read:

“The authors thank the help from the Statistical Analysis Laboratory, Department of Internal Medicine and the Statistical Analysis Laboratory, Department of Medical Research, Kaohsiung Medical University Hospital. This study is based in part on data from the National Health Insurance Research Database provided by the National Health Insurance Administration, Ministry of Health and Welfare and managed by the Collaboration Center of Health Information Application (CCHIA), Ministry of Health and Welfare, Executive Yuan, Taiwan. The interpretation and conclusions contained herein do not represent those of Ministry of Health and Welfare. This work was supported by grants from the Ministry of Science and Technology [MOST 104-2314-B-037-005 and MOST 104-2314-B-037-034-MY3]; the Aim for the Top Journals Grant, Kaohsiung Medical University Research Foundation [KMU-DT103008] and Kaohsiung Medical University Hospital [KMUH104-4R07].”

